# Around the Hospitals

**Published:** 1894-02-10

**Authors:** 


### AROUND THE HOSPITALS.

Notice to the Managers of Institutions.—Special space is now reserved for the insertion of notices of hospital meetings and festivals. The Editor requests that all notices may reach The Hospital Office, 428, Strand, at least one week before the date of each meeting.

**Ayr County Hospital.**—This hospital, which presents a report admirable in many respects, is able to state that whilst the percentage of deaths generally is as low as 5·25 per cent., that of amongst surgical patients is still more markedly so, and amounted to little over 3 per cent. Many improvements have been effected during the year. The drainage and sanitary works necessary have been entirely renewed, the laundry accommodation and disinfecting apparatus have been rendered complete and efficient, and the nurses' quarters have been improved and added to. Although not quite so satisfactory as last year, there is still little to complain of in the financial condition of the Ayr Hospital. The weak spot, namely, congregational and working men's contributions, will receive especial attention during the coming year, and then, doubtless, all necessary funds will be secured. A fair number of legacies, amounting to £1,118, have been placed to the account of the endowment fund. The list of donations for special purposes, such as repainting of particular wards, shows ability on the part of the management to bring out the wants of the moment. The larger expenditure which the accounts for the past year show are accounted for by the fact that the number of scarlet fever patients was unusually large, causing additional expenditure for maintenance and nursing. This cost is not likely to re-occur, as arrangements as to fever patients are being made with the Local Board.

**The Belfast Hospital for Consumption.**—The annual report of this hospital brings out two facts very strongly, firstly that the utmost use is made of the opportunities offered to patients, and secondly that the assistance rendered is out of all proportion to the needs of the community. The enormous percentage of sufferers from consumption, as compared with other diseases, in the British Isles would hardly seem to be realised generally. At Belfast the support afforded the Consumption Hospital is not only inadequate for the needs of consumptive poor in the city needing hospital care, but it is insufficient to maintain the small number of beds which could be used at that institution without incurring debt. Through the untiring exertions of those interested in the hospital, a house adjoining the premises has been acquired and fitted up as an additional temporary wing, and it is to be hoped that this extension of the work will awaken further interest, prove how eagerly available accommodation is sought for, and finally lead to a really adequate and completely equipped new hospital being secured for the sufferers from this terrible disease before very long.

